# Emerging viral threats and the simultaneity of the non-simultaneous: zooming out in times of Corona

**DOI:** 10.1007/s11019-020-09970-3

**Published:** 2020-07-31

**Authors:** Hub Zwart

**Affiliations:** Dean Erasmus School of Philosophy, Erasmus University Rotterdam, Bayle Building/Room J5-65/Burgemeester Oudlaan 50, 3062 PA Rotterdam, The Netherlands

**Keywords:** Virology, Viroscience, Globalisation, Emerging viral threats, Simultaneity of the non-simultaneous, Dialectical materialism, Marxist bioethics, Global bioethics

## Abstract

This paper addresses global bioethical challenges entailed in emerging viral diseases, focussing on their socio-cultural dimension and seeing them as symptomatic of the current era of globalisation. Emerging viral threats exemplify the extent to which humans evolved into a global species, with a pervasive and irreversible impact on the planetary ecosystem. To effectively address these disruptive threats, an attitude of preparedness seems called for, not only on the viroscientific, but also on bioethical, regulatory and governance levels. This paper analyses the global bioethical challenges of emerging viral threats from a dialectical materialist (Marxist) perspective, focussing on three collisions: (1) the collision of expanding networks of globalisation with local husbandry practices; (2) the collision of global networks of mobility with disrupted ecosystems; and (3) the collision of viroscience as a globalised research field with existing regulatory frameworks. These collisions emerge in a force field defined by the simultaneity of the non-simultaneous. Evidence-based health policies invoke discontent as they reflect the normative logic of a globalised knowledge regime. The development of a global bioethics or macro-ethics requires us to envision these collisions not primarily as issues of benefits and risks, but first and foremost as normative tensions closely entangled with broader socio-economic and socio-cultural developments.

## Introduction

In response to the Coronavirus (COVID-19) pandemic, the global political economy faces a dramatic moment of suspense (involving hundreds of millions of people) whose tumultuous impact materialises into a “new normality”. The world will never fully reset to “normal” neo-liberalism. In this paper, the COVID-19 pandemic will be regarded as symptomatic for the current era of globalisation and hyper-connectivity: a more or less inevitable consequence of the way in which the global socio-economic system has evolved during recent decades. The Corona-crisis seems to expose a *series* of (environmental, biological, economic, occupational, political and ideological) crises already evolving for quite some time, but now converging into a major disruptive transition. To bring this to the fore, this paper adopts a dialectical materialist (Marxist) bioethical framework, focussing on the socio-cultural (“super-structural”) dimension. Viral diseases are part of an ongoing transition that began during the industrial revolution, transforming agricultural societies into a high-tech, world-spanning agro-economic system. A dialectical framework emphasises the connectedness of global bioethical challenges surfacing in the context of COVID-19 with ongoing developments in adjacent areas such as environmental degradation, animal husbandry, food production, global mobility, and technoscientific data management.

On the level of virology as such, a discourse has emerged which sees viral diseases as symptomatic for the extent to which humans have evolved into a global species (Wald 2008; Caduff 2014, 2015a). After decades of “viral optimism” (Garrett 1994), emerging viral diseases resurged as a public health concern in 1981, when the first *AIDS* cases were reported and HIV entered the global scene. The global community entered a new (post-modern) historical constellation, and viral threats were part of this emerging scene. The Coronavirus (COVID-19) outbreak, which was officially proclaimed a pandemic by the *World Health Organisation* (WHO) on Wednesday March 11 2020, is a health crisis which first of all incites us to *zoom in*, focussing on concrete policy measures to be adopted to flatten the proliferation curve and keep the number of affected patients manageable. Yet, from a dialectical and global bioethical perspective, the crisis also summons us to *zoom out*, considering the broader socio-economic and socio-cultural context in which this disruptive event unfolds. Whereas most other scientific disciplines (from virology up to economics and management studies) inevitably zoom in on concrete developments and dilemmas entailed in the COVID-19 outbreak, a global bioethical analysis rather urges us to see the Coronavirus challenge as a case history emerging against the backdrop of an extended temporal horizon.

Against this *general* (socio-economic) backdrop, I will focus on three *particular* instances of moral (super-structural) collision, each of them connected with *concrete* viral threats. First, I will analyse the collision between expanding networks of globalisation and local husbandry practices. Subsequently, the focus shifts to the collision of global networks of mobility with disrupted ecosystems, resulting in viral threats for humans as a global species. Finally, I will address collisions involved in viral research as such, focussing on moral issues entailed in handling sensitive and valuable bio-information, underscoring how virology evolved into *viroscience*: a globalised, high-tech research enterprise, challenging established cultural and governance practices. Before analysing these collisions more systematically, however, I will present the backdrop of my analyses. First of all, I will outline my conceptual framework, via a short history of human-pathogen interactions seen from a dialectical materialist perspective. Subsequently, I will provide a short historical overview of viral discourse and its vicissitudes.

## From *Common Human Pattern* to *Global Village*: a dialectical materialist view of pandemic challenges in human history

The dialectical materialist concept of history, as originally developed by Karl Marx and Friedrich Engels, assesses the present against the backdrop of an extended historical process in which dialectical patterns can be discerned, i.e. series of transitions from initial situations of relative stability (the first moment), which are challenged by disruptive events (the moment of negativity or contradiction) until a new equilibrium is reached on a higher level of complexity (the “negation of the negation”). As Marx argued in *Capital* (1867/1979), during the industrial revolution, capitalism disrupted and “negated” the “rural communism” practiced in more or less self-sufficient villages in the pre-industrial past (Engels 1880/1962, p. 215), so that farmers were expropriated and forced to migrate into industrialising urban areas, where struggle for existence raged (Engels 1880/1962, p. 216). This involved, among other things, the estrangement of production and consumption, as commodified food products were no longer produced collectively by consumers themselves (in villages), but in factories, as commodities, so that consumers from now on had to buy these food products (e.g. industrially produced bread, beer, canned meat, etc.) on the market (Zwart 2000, 2005). Traditional agricultural and artisanal know-how was replaced by scientific knowledge (mathematics, chemistry, logistics, human resource management, etc.) to rationalise and increase the pace and scale of the food production process. Yet, Marx and Engels argued, although industrial production *seemed* rational, it actually resulted in anarchy and contradictions (e.g. highly competitive food markets, environmental pollution, massive waste, social disruption, etc.).

In the course of the twentieth century, this basic conception which, according to Marx and Engels, not only applies to European history but to other continents as well (Engels 1884/1962), was further elaborated by a considerable number of Marxist scholars. According to Marxist historians Romein and Romein (1954), for instance, during the Neolithic revolution (between 10,000 and 5000 years ago), agriculture gave rise to a “Common Human Pattern” (CHP), a wide-spread and relatively stable agricultural life-style, practiced in small-scale, self-supporting villages throughout the human world (the first moment). According to these authors, the basic features of CHP village life were remarkably constant and relatively independent of the political context and the exploits of the political elite. This life-style was disrupted, however, by the onset of the Industrial Revolution in modern Europe (the second moment), as documented by Marx in *Capital* (1867/1979). Two revolutions, the Neolithic and the Industrial one, put the CHP between parentheses as it were (Zwart 2009a).

In Europe during the CHP, humans and domesticated animals lived in close proximity, often under the same roof, interacting with one another intensively on a daily basis. The CHP evidently entailed metabolic challenges of its own. Activities such as feeding, milking, slaughter and cleaning stables were carried out virtually unprotected, bringing rural human populations in regular contact with saliva, milk, blood and excrements of their animals. Thus, a plethora of infectious diseases affecting human populations emerged during the past ten millennia or so, following the rise of agriculture (Wolfe et al. 2007). As a result, human populations were exposed to, and gradually became immune to, pathogens coming from livestock (with group immunity representing the “negation of the negation”, the third moment). During periods of migration or conquest, human invaders from agricultural homelands functioned as carriers of infectious diseases for which they themselves had become resistant, exporting the disruptive “negativity” of viral and microbial threats to other continents. A notorious example is the conquest of what is now Latin America by relatively small bands of conquistadors, resulting in the rapid collapse of Aztec and Inca civilisations. As Diamond (1997/2005) emphasised, infectious diseases were responsible for the decimation of native populations on a massive scale and therefore a major factor in these events.

As Marxist historians Romein and Romein (1954) phrased it, during the Industrial Revolution (the second dialectical moment), the modern Western world began to “deviate” from the CHP. This notably gave rise to large industrial centres, either by expanding and transforming (“industrialising”) existing cities such as Paris, London and Vienna, or by creating new urban centres, such as Manchester or New York. As Marx argued in *Capital*, while rural communal production systems were systematically devastated, large numbers of humans were forced to migrate from rural to urban areas, where hygienic conditions were often appalling (Engels 1845/1962; Marx 1867/1979; Foster 2000). Friedrich Engels was highly sensitive to environmental and public health aspects from the very outset. Modern industry’s most decisive “product”, besides the working classes themselves, Engels argued, were big booming cities such as Manchester (1845/1962, p. 237, p. 250, p. 254). He acutely described its miasmic air, hideous smell (p. 259) and polluted puddles (p. 274) such as the river Irk, which had become a narrow, coal-black, foul-smelling stream, filled with refuse and excrements (p. 282). Such urban dwellings created optimal conditions for the spread of infectious diseases such as cholera, Engels argued (p. 295). Thus, as urbanisation proliferated, the identification of pathogens became an acute scientific challenge and the 1880s became the heydays of “microbe hunters” such as Louis Pasteur and Robert Koch (de Kruif 1926),—a confirmation of the entanglement of scientific progress (the birth of microbiology) with socio-economic conditions. Dialectically speaking, microbiology represented a “negation of the negation”: a scientific (super-structural) attempt to restore immunity and stability at a higher level of urbanisation and productivity (the third moment). Rather than representing the “end” of history, however, this new situation inevitably entails multiple instances of disruption, unevenness and negation of its own.

During recent decades, due to new methodologies and insights emerging in a broad array of research fields, the scope of the dialectical materialist approach was significantly broadened, thereby becoming more sensitive to global diversity, differentiation and overdetermination, while casting off the teleological legacy of bourgeois progress-speak (seeing human history as a global socio-economic theatre where the survival of the fittest was enacted). First of all, more attention was given to living conditions in pre-agricultural societies. Engels (1884/1962) already highlighted the “primitive communism” of pre-agricultural communities of gatherers and hunters, a line of research which was taken up by authors such as Sahlins (1972), emphasising the affluent communism of “stone-age economics”. Harris (1979) coined the phrase “cultural materialism” for a science of culture that relied on, but also expanded the approach originally developed by Marx and Engels. Starting point for understanding social change was a triadic distinction between *infrastructure* (technology, economics, production), *structure* (socio-cultural organisation) and *superstructure* (ideology, religion). The disruptive impact of Europe’s expansion on other continents became a crucial theme in this genre of writing.

A further significant broadening of the horizon resulted from the increased awareness of the decisive importance of *biological* factors, besides technological, socio-economic and socio-cultural ones. To understand the present, we must significantly broaden our temporal horizon and learn to think in terms of “deep” evolutionary time. As Engels argued, the whole of geology is an extended series of dialectical transitions (1878/1962, p. 127), greatly affecting the conditions for life on earth. An important turning point occurred ~ 175 million years ago, when the primordial supercontinent Pangaea (“one earth”) began to break apart and a single, all-encompassing landmass was sliced into two separate halves: Eurasia and the Americas, where distinct evolutionary pathways evolved, until an expedition led by Christopher Columbus set foot on a Caribbean island in 1492. The “Columbian exchange”, as historian Crosby (1973/2003) phrased it, reknit the seams of Pangaea and unleashed a process of biological levelling. Not only humans, weapons and ideas, but also a whole menagerie of plants, animals, microbes and viruses, food crops and diseases (small pox, syphilis, etc.) began to spread across the continents. The physical reintegration of previously separated ecosystems created a new material base, resulting in the homogenization of biological life, a process which is still ongoing (Crosby 1973/2003; Mann 2011; Nunn and Qian 2010). In dialectical terms, Pangaea represented the first moment of primordial unity, giving way to negation and differentiation through the formation of separate continents where particular suits or flora and fauna began to evolve (the second moment), until the process of modern globalisation gave rise to a sudden reunification (a negation of the negation), with human migrants and human technologies acting as carriers of connectivity (the third moment).

Against this backdrop, a genre of literature emerged which emphasises the role of infectious diseases in world history. Diamond (1997/2005) was already mentioned, whose best-selling book describes how European conquistadors acted as vectors for pathogens to which they themselves had become immune, so that microbes became a decisive factor in the process of colonisation, although in the case of malaria and yellow fever, germs could also present an obstacle to Western expansion. Brian Fagan in *Clash of Cultures* (1984) likewise documented how globalisation, besides cultural and socio-economic disruption, brought epidemic diseases to other continents. A landmark publication in this genre was William McNeill’s *Plagues and Peoples* (McNeill 1976), examining the impact of infectious diseases throughout the ages. Contrary to hopes and assumptions that had been spawned by the dawn of the antibiotic era (progress-speak), there was now a growing awareness of how plagues and people remained inextricably linked. As Sommerfeld (2003, p. 532) phrases it, this growing awareness of the interrelatedness of biotic and social factors resulted in a more comprehensive approach, examining the broader context of infectious diseases by paying special attention to macro-economic and socio-political tensions. As a result of macro-economic globalisation, studying the connectivity between societal developments and infectious disease became more relevant than ever.

Thus, besides dramatically affecting landscapes in the West, capitalism fuelled processes of colonisation and globalisation. The industrial revolution as a socio-economic “deviation”, together with its biological correlates, expanded into other areas and continents, adding a global dimension to the challenges of “microbe hunting”. A symptomatic disruptive event was the “second” (1826–1937) cholera pandemic, originating in India and spreading into Europe,^1^ the Americas, China and Japan as the “classic epidemic disease of the nineteenth century”, intimately connected with colonialism (Arnold 1986) and challenging the ideology of global progress. Marxist author Maxim Gorky wrote a telling cholera play, set in a family mansion owned by a wealthy amateur chemist named Protasov, who firmly believed that progress in chemistry (more specifically: *his* research) would bring about a better world (Gorky 1905/2013). When a cholera epidemic breaks out, suspicion quickly spreads among villagers that the disease was either caused by physicians trying to create lucrative activities for themselves, or originated in Protasov’s home-made laboratory, as chemical run-off from an unsafe storage tank polluted the water supply. The play ends with an angry mob storming Protasov’s mansion (representing the life and views of a self-centred, privileged, bourgeois elite).

As industrialisation and urbanisation evolved from a Western “deviance” into a global mainstream phenomenon, global society became “one world” (Singer 2002), both economically and biologically, with biological connectivity and environmental devastation as two sides of the coin. Whereas according to Marxist authors the CHP’s metabolism between humanity and nature remained relatively sustainable, the industrial revolution gave rise to an “ecological rift” (Foster et al. 2010), to massive processes of disruption (environmental pollution, soil degradation, urbanisation, alienation) which dramatically aggravated during the current era of globalisation. These developments fuelled a revival of interest in Marxist approaches to the current ecological crisis (Foster 2000; Fuchs and Mosco 2015a; Moore 2016), underscoring the detrimental environmental and biological impact of our global economic system.^2^ Seen from this angle, emerging viral threats represent a symptom of excessive human estrangement from nature. While during the CHP the production and consumption of food and other commodities were closely interconnected processes, in the global agro-economic system of today the distance between production and consumption has dramatically increased (Zwart 2000), resulting in multiple symptoms of alienation: from environmental degradation via massive food waste up to wide-spread consumer distrust (Korthals 2010). This backdrop explains why during the twentieth century viruses became an issue of concern and why virology evolved into such a prominent research field.

## The birth of viroscience and the outbreak narrative

In 1898, more than a decade after the discoveries of Pasteur and Koch, Martinus Beijerinck discovered the “virus”, heralding the beginning of virology.^3^ Viruses are uncanny entities: sets of genes encased in proteins; faceless, semi-biotic and invisible (Braun 2007), swarms of ephemeral creatures caught in constant flux (Caduff 2015b). In the course of the twentieth century, microbiology and virology generated effective measures to counteract microbial and viral threats, notably by improving hygienic conditions and developing vaccines. During the 1950s and 1960s, polio and smallpox seemed about to be eradicated and in 1967, the U.S. Surgeon General stated that “the book on infectious diseases” could be closed (Garrett 1994), suggesting that, *thanks to virology*, disruptive biological threats had been effectively addressed and a new epoch of stability could now evolve at a higher level of socio-economic complexity.^4^

As the first AIDS cases were reported, however, viral threats resurged as issue of concern. In 1994, two best-sellers were published by science authors Laurie Garrett (1994) and Richard Preston (1994) inaugurating the viral “outbreak narrative” (Caduff 2014, 2015a), staging the vulnerable West as threatened by an obscure mix of dangerous pathogens lurking in remote areas: a recoil of colonisation, globalisation and ecological devastation. This genre of discourse sees viral diseases as symptomatic for the current human condition. Rapid population growth, combined with massive urbanisation, notably in regions in Africa, Asia and Latin America, in combination with global connectedness and mobility (of humans and accompanying species), but also climate change, environmental and ecological disruption, deforestation and the destruction of previously pristine habitats: all these factors became facilitators for the rapid spread of potential pandemics from confined populations to global dispersions. Viruses became actors on a global stage, co-determining our future, as viral threats became interconnected with demographic, technological and socio-cultural transitions. As human activity acquired a pervasive and irreversible impact on the global environment, urban centres interconnected into a global village (Hamilton 2017). Paradoxically, while human health has significantly improved during recent decades, environmental deterioration poses new health threats (Mackenbach 2007; Ten Have 2016, 2019), paving the way for potential pandemics. New viruses may not only come from tropical regions, moreover, but may also be released by Siberia’s melting permafrost, for instance, where smallpox viruses may be set free.^5^

Already during the early 1990s, Stephen Morse called attention to the extent to which global human mobility, as an inherent dimension of the global economy, precipitated the emergence of new viral threats (Caduff 2015a, b; Morse and Schluederberg 1990; Morse 1990; Morse 1995). As a global species, Morse argued, humans facilitate viral traffic by establishing global viral highways on an unprecedented scale, in combination with other disruptive developments such as deforestation and massive urbanisation, notably in quickly expanding megacities in the global South. Similar to how in the 1880s (during the era of Pasteur and Koch) modern society became aware of the prominent role of microbes in the environment,—both beneficial and harmful to human existence (de Kruif 1926)—, humanity now becomes increasingly aware of the prominent role of viruses as global agents.

Thus, viruses became a global human health risk (Claas et al. 1998; Marston et al. 2014). Besides novel coronaviruses (SARS-CoV, MERS-CoV, COVID-19), influenza viruses (H5N1, H1N1, H7N9), bunya-viruses and henipaviruses, global society is confronted with the spread of pathogens that were previously confined to tropical regions, such as Dengue and Zika viruses. Zoonosis (i.e. the transmission of infectious diseases from animals to humans) plays a key part in transmission. As David Quammen phrased, “zoonotic spillover” (viral transfer from animal hosts to humans) is “a word of the future, destined for heavy use in the twenty-first century”, representing “the most significant growing threat to global health” (Quammen 2012, p. 21). Although viruses are quite diverse, they are grouped together as “emerging viral threats”, representing a sinister, semi-living shadow, accompanying humans as they traffic around the globe, confronting us with an unsettling paradox. On the one hand, humans as a global species affect and disrupt the conditions for life on earth on an unprecedented scale. Via technoscience, “we” increasingly control the planet. And yet, there is a disquieting sense of powerlessness as well, notably when it comes to developing a convincing societal response to the disruptive consequences of our dominance. This paradox is noticeable in debates on climate change, mass extinction and the governability of CRISPR-cas9, but it also affects deliberations concerning emerging infectious agents. In other words, while our technoscientific prowess continues to expand, our capacities for macro-ethical and political action (to contain the disruption and restore the balance) develop at a non-simultaneous pace.

Emerging viral diseases entail a plethora of normative challenges. While viral research has become a global enterprise producing enormous amounts of data, this does not necessarily empower global capacities for action. While rapid identification of emerging viral diseases and the provision of timely insights into their origins, modes of transmission and clinical impacts are acknowledged as important items on the global health agenda, and while specialised laboratories are using *Next Generation Sequencing* (NGS) techniques as powerful tools for virus discovery (metagenomics) and for developing tailored options for diagnostics, prognostics and therapy (*theranostics*), virology actually reveals (more than ever) how vulnerable we are. As Braun (2007) formulated it, virology makes us acutely aware of the extent to which humans are vulnerable beings, thrown into an unpredictable molecular world, a global web of circulation and exchange, haunted by the spectre of newly emerging or unspecifiable risks: the great “biological cauldron” of the twenty-first century. Via international trade, traffic of livestock and food and other forms of connectivity, such as air travel, tourism and migration, the outbreak narrative suggests that the biological existence of people in Singapore or Toronto has become intimately connected (in real time) to the lives of wild bats in China or domesticated ducks in Vietnam.

Against this general backdrop, I will now focus on three particular collisions, each of which will be illustrated with the help of concrete (specific) viral threats, beginning with the collision between expanding globalised food production networks and local husbandry practices, many of which continue to reflect basic features of the CHP, including intensive, unprotected interaction with animals. It has been claimed, for instance, that SARS (the first emerging infection of the twenty-first century) originated in a duck pen in China, from where it quickly entered the global arena (Wald 2008) and that COVID-19 originated at a “wet” animal market in Wuhan. Subsequently, I will discuss the collision of global networks of mobility and traffic with disrupted ecosystems, notably in tropical Africa, giving rise to emerging infections such as Ebola. The third and final collision emerges between viroscience as a globalised research field with local and national practices, focussing on the rapid spread of sensitive information about viral threats (produced by NGS research) and the ability (or inability) of existing or emerging ethical, legal and policy frameworks for responsible data management to deal with this (as exemplified by the case of H5N1, the bird flu virus, potentially lethal for humans as well).

## Collision 1: traditional husbandry practices challenged by globalisation and vice versa

Since the agricultural revolution, humans and domesticated animals (cattle, horses, camels, dogs, cats, etc., but also the microbes and viruses associated with them) lived closely together in symbiotic, zoonotic and immunising ecosystems. Currently, however, agriculture is rapidly evolving into a high-tech, highly specialised enterprise, while rural populations migrate to quickly expanding urban centres. Comparable to what happened in the nineteenth century (when Marx wrote *Capital*), the distance between production and consumption of food products has increased dramatically, with farm animals being concentrated within specialised facilities. We no longer live under one roof.

Yet, traditional animal husbandry practices continue to exist. Two different historical epochs or ways of life coexist side by side: the CHP and the life-style of globalisation, mutually affecting and challenging one another. They are, as Maxim Gorky (1919) phrased it, like two incompatible “civilisations”, increasingly close to one another, but evolving at a different pace. In the context of the global village, past and present, traditional isolation and global connectivity share the same planetary environment. In Marxism, this phenomenon is known as the “simultaneity of the non-simultaneous” (“Gleichzeitigkeit des Ungleichzeitigen”: Gorky 1919; Pinder 1926; Bloch 1932/1977; Jameson 1991). Not all people exist in the same Now, not all life-styles adhere to the same pace and rhythm. There are many regions in the world where pre-industrial proximity between humans and domesticated animals is still quite intense. Davis (2006) sees southern China for instance, where huge numbers of pigs, domestic ducks and wild waterfowl live in traditional ecological intimacy with humans (p. 17) as a potential cradle for pandemics. Processes such as described by Marx in *Capital* (industrialisation, urbanisation, accumulation) are now happening right there (p. 13). This simultaneity (of the pre-industrial past and the global transportation networks of the present) entails disruption on both sides. While traditional life-forms are under the pressure of globalisation, local instances of zoonosis may rapidly enter global networks and assume a global impact. Therefore, as Marx pointed out, global capitalism displays the tendency to impose simultaneity (synchronicity) at the expense of geographical and cultural diversity (Harvey 2001). Against the backdrop of socio-economic globalisation, non-simultaneity is considered a problem, giving rise to symptomatic anxieties about mysterious microbes breeding in remote jungle villages or urban slums, where humans are living in close proximity to their backyard ducks and rooftop poultry (Caduff 2014). In dialectical terms: now that the pre-modern past has been rendered obsolete by globalisation, and in order to regain a technically advanced situation of global stability, non-simultaneity becomes a *contradiction*: something which must be overcome.

MERS provides an interesting case study in this respect. In 2014, the WHO announced that the *Middle East Respiratory Syndrome* (MERS) was caused by a novel coronavirus (MERS-CoV), first identified in Saudi Arabia in 2012. Transmission occurred from camels to humans (McGrath 2014; Memish et al. 2014; Funk et al. 2016). In response to this threat, the Saudi government launched a campaign to discourage eating raw camel meat or drinking unpasteurized camel milk. People were urged to wear masks and gloves when dealing with camels and to avoid sick animals. This invoked a cultural recoil, however, with camel drivers demonstratively hugging and kissing animals in front of tourist cameras, in defiance of the viral threat: an emphatical enactment of the simultaneity of the non-simultaneous. Techniques of globalisation, advocated by scientists and governments in a top-down manner (e.g. wearing mouth masks and gloves when dealing with animals) collided with local husbandry practices, resulting in symbolic gestures (enacted before a global audience of tourists), emphatically endorsing the type of close physical interaction characteristic of the CHP. From a Marxist perspective, such collisions demonstrate the importance of “superstructure” (i.e. culture). Camels no longer play a significant economic role in Saudi Arabia outside tourism. What is disrupted and challenged by the logic of globalisation are cultural (super-structural) patterns, values and identities.

Similar instances of “super-structural” recoil can be discerned in the context of food production. Food-borne viruses play a significant role in the transmission of infectious disease (Koopmans and Duizer 2002; Koopmans et al. 2002). This not only applies to raw food (e.g. shellfish), but to all food products handled manually. Although the possibility that the COVID-19 outbreak was caused by laboratory manipulations of SARS-CoV-like viruses in vitro is lively debated (Andersen et al. 2020), the consensus view is that it can be traced back to a “wet” animal market in Wuhan, visited by humans, but also by rodents and cats, where not only fish and shell fish, but also various species of exotic animals are for sale. Such practices reflect super-structural rather than economic values. Adaptation to tested policies of sanitation will increase safety, but faces cultural resistance: discontent in industrialised practices of food handling and consumption. Thus, rather than arguing that “base determines superstructure” in a deterministic fashion, such collisions reflect the mutual tensions that may arise between infrastructural transitions (globalisation) and “non-simultaneous” socio-cultural practices.^6^ As Koopmans and Duizer (2002) phrases it, any food item processed manually is a possible source of infection, while contamination may occur at almost every step in the path from farm to market to table, so that local food-borne epidemics may diffuse into international food-borne outbreaks on a globalised food market (Verhoef et al. 2011). Globalisation implies maximised distance between production and consumption, as food products are distributed to geographically distant locations, problematising practices which involve humans physically *touching* food. Compulsive hand washing, once considered a neurotic obsession, becomes part of standard protocol, not only in hospitals, but also in food industries, restaurants and supermarkets (i.e. places where infected individuals may become super-spreaders). Eventually it becomes the new normal also in private homes.

The distance between production and consumption necessitates interventions for making the food chain safer. To address the vulnerabilities of globalised food circuits, various techniques for prevention and surveillance have arisen. Besides practical measures such as washing hands or wearing disposable gloves, this may involve high-tech biomolecular or NGS methods for tracing potential viral threats. DNA sensors may be installed, not only in hospitals and airports, but also in food production facilities and markets. From an experiential perspective, by introducing such methods, what used to be familiar and intimate (the daily handling of food) becomes a target of suspicion and estrangement (Zwart 2015). A cordon sanitaire is imposed between humans and their food, inciting discontent in global civilisation, as traditional food handling practices of long standing are reframed as problematic. Thus, cultural tensions reflect socio-economic changes which (often quite suddenly) label established practices (e.g. in animal husbandry and food processing) as untenable (“non-simultaneous”). The simultaneity of the non-simultaneous should not be considered a one-directional process, inevitably heading towards modernisation, however. A de-industrialisation of animal husbandry may well be part of a more sustainable future, as predicted by Marx and Engels (Engels 1878/1962, p. 276; Foster 2000), who argued that sustainability requires a sublation of the antagonism between urban and rural areas.

The distance between agricultural production and consumption implies that large numbers of domesticated farm animals spend their lives in specialised facilities, often completely invisible for consumers, where they are kept, fed and slaughtered by experts (wearing white coats or plastic coveralls and gloves): a condition (thematised by Marxism as estrangement) which became endemic after the disruption of the CHP. Paradoxically, however, opposite trends can be discerned as well, as urban life-worlds become densely populated by pets. Increasingly, besides cats, rodents and dogs, this involves commodified exotic pets (and the micro-organisms and viruses accompanying them). In the global village, interaction with household pets can be very intense, as humans cuddle their animals or allow them to share their bed (“zoonosis in the bedroom”, Chomel and Sun 2011), resulting in “risky sleeping arrangements” (Braun et al. 2015). Again, zoonosis is more than a purely biological threat, to be countered by implementing hygienic policies, but reflects socio-economic and socio-cultural transitions, not only the dramatic increase of the distance between animal husbandry and food consumption, but also the emergence of global pet markets and the invasion of commodified consumer pets in urban settings.

## Second collision: global mobility and disrupted ecosystems

By reducing spatial distance, globalisation not only increases proximity of global networks to traditional husbandry practices, but also to areas which, until recently, were regarded as relatively pristine, such as rain forests in Central Africa, where HIV originated. Disrupted ecosystems are exposed to processes of globalisation, so that lethal viruses, “hiding” in rainforests, may suddenly “leak” into the networks of global interconnectivity, turning them into conduits for communicable diseases. The “outbreak narrative” (Wald 2008) involves a latent viral threat which suddenly evolves into a global pandemic, via air traffic and other means of global transport and communication. This narrative is not only discernible in academic papers and policy reports, but also in virus novels (Wald 2008; Zwart 2019) as a formulaic plot, commencing with the identification of an emerging infection (somewhere in tropical Africa), from where it is transmitted through global networks, while viral experts and global health organisations (e.g. the WHO) are called upon to design containment policies. In reality, the route of transmission may well be in the opposite direction, e.g. the spread of HIV via global networks to African countries, but the outbreak narrative is biased towards one-directional spread, reflecting the dominant view that deadly diseases emerge in the South or East, gaining momentum in the mega-slums of Asia and Africa, from where they spread into the West (Caduff 2014). Thus, non-simultaneity is framed as a one-sided process, while in reality we witness complex forms of interpenetration.

The outbreak narrative also entails the idea that Nature is striking back: framing *us* as the invaders, while Planet Earth is mounting an immune response (Wald 2008). Via lethal viral parasites, the earth’s immune system is fighting us off, so that emerging infections are a response to our venturing into primordial places which should have been left undisturbed: the recoil of human trespassing via a plague.^7^ The moment of truth of this narrative script is that we are facing the detrimental results of a disrupting “metabolic rift” (Foster 2000) caused by socio-economic expansion on a global scale. This second collision pictures humanity itself as a global species under threat. In his dialogue *Statesman*, Plato already voiced the idea that governance amounts to “human husbandry”: the management of humankind (for instance with the help of containment measures such as lock-downs). Politics, Plato famously argued, is art of tending the human herd (Plato 1995, 267C) and an infra-structural lock-down can be considered an “anthropo-technique” for safeguarding the health of humans as a self-domesticated species (Sloterdijk 1999/2001; Zwart 2009b).

A telling exemplification of the second collision is the 2014 outbreak of Ebola Virus Disease (EVD), a viral haemorrhagic fever caused by ebolaviruses. Ebola spreads through direct contact with body fluids (blood, saliva, mucus, vomit, faeces, sweat, tears, breast milk, urine, semen) of persons who developed symptoms of the disease. Zoonosis (notably involving bats and primates) played a role as well. The largest outbreak to date was the epidemic in West Africa from December 2013 to January 2016, claiming a death toll of more than 11,000 victims (while nightmare scenario’s forecasted millions of deaths). In the case of Ebola, various instances of non-simultaneity can be discerned. First of all, as technoscientific research areas such as viral genomics developed into a global phenomenon, bioethics aims to globalise as well, so as to safeguard normative values such as fairness and autonomous consent, but the global validity of bioethics remains an issue of dispute (Levitt and Zwart 2009, p. 371), notably its sensitivity to socio-cultural contexts, especially in regions where bioethical infrastructures are less robust. Alenichev and Nguyen (2019) point out how principles of Western research ethics failed to satisfactorily address the normative challenges involved in conducting Ebola vaccine trials in West Africa, where participation by healthy volunteers was compensated by payments, food packages and certificates, so that research subjects experienced their participation as labour. From their perspective, vaccine research constituted a job market created by powerful international research enterprises. Participation by (mostly illiterate) subjects was encouraged by the absence of other stable forms of income, but discouraged by rumours that Ebola researchers were wilfully spreading the disease. In short, a practice which at first glance constitutes a legitimate form of data collection, may on closer inspection be regarded as “slum” research, involving the exploitation of an illiterate workforce by wealthy global actors (the enterprise of global technoscience and digital capitalism).

Another example of non-simultaneity was the conflict revolving around traditional burial practices (e.g. ritual washing of the deceased) which, according to Western experts, significantly contributed to the spread of Ebola. Global organisation such as the Red Cross tried to replace them by “safe and dignified burials” (Bah and Aljoudi 2014; Tiffany et al. 2017). Based on global viroscientific insights, traditional healing and burial practices were branded as unsafe. From a Marxist bioethical (*macro*-ethical) perspective, such collisions indicate how socio-economic transitions (globalisation) problematise cultural (“super-structural”) practices, up to enforced replacement or sanitisation of time-old rituals.

The collision between local practices (ritual burials) and global practices (sanitation of interactions) is symbolised by disposable items such as condoms, mouth masks and medical gloves, which are introduced to promote hygiene by creating and maintaining physical boundaries, especially in contexts where close proximity used to be the rule. When dealing with sexual partners (HIV), domesticated animals (MERS) or corpses (Ebola), individuals are forced or encouraged to adopt protected forms of interaction: safe sex, safe animal husbandry and safe burial practices, symbolised by disposable hygienic aids. Thus, the *quantitative* intensification of contacts between humans across the globe has significant implications for the *qualit*y of human interactions, as valued forms of physical contact must be redesigned. This may collide with the perceived symbolic or experiential value of physical proximity for the practices concerned. Concepts such as “clean” and “dirty” (and sanitary rituals connected with them) are historical variables (Douglas 1966). In the face of emerging viral threats, such super-structural signifiers are rapidly being redefined.

## Third collision: global information networks and mechanisms of defence

The role of viral research in the global landscape remains ambiguous. Viroscience aims to make the world safer for humans by signalling and addressing viral threats, but by accumulating and circulating terabytes of information concerning emerging and potential dangers, our sense of safety may actually decrease. Equipped with superfast and hyper-precise NGS technologies, viroscience provides high resolution insights, for instance concerning the zoonotic risks involved in food processing or human-animals interactions. Inconvenient messages label traditional practices as non-simultaneous compared to dominant globalisation trends. Technologies for high-throughput sequencing were developed as laboratory tools, but now we witness their performance outside laboratories, under “messy” circumstances, also from a super-structural (e.g. socio-cultural and bioethical) viewpoint. In the terabyte era, extramural socio-cultural ambiances become living laboratories, where technoscience generates and circulates overloads of bio-information concerning potential dangers.

The technoscientific quest for viral bio-information purports to make decision-making more evidence-based, but also transforms the public sphere. Strategic locations (e.g. airports, hospitals, the food industry, etc.) become equipped with smart virus detectors collecting massive amounts of data about human individuals and the viral life forms they carry with them. Surveillance technologies keep track of the biomolecular trails (DNA finger-prints, specks of biomolecular dust) we leave behind, giving rise to an NGS-based panopticon continuously tracking us and increasingly relying on app-based practices of biomolecular self-portrayal, self-monitoring and self-surveillance, resulting in a “molecularised me” (Zwart 2018b), urging us to redefine ourselves in terms of bio-molecular vulnerabilities and susceptibilities. NGS technologies represent virologic globalisation: forms of research conducted by global networks of laboratories whose outcomes are published and shared via digital repositories. Viroscience evolved into a global enterprise, exemplifying what has been thematised as “information capitalism” (Ignatow 2017) or “surveillance capitalism” (Zuboff 2019), i.e. the commodification of data on a global scale, in the context of the global knowledge economy, where production and circulation of data is becoming a more important source of value than material production, giving rise to new forms of social inequality (the digital divide). Whereas from a global viroscientific perspective the availability of massive viral data is a requirement to effectively address emerging infectious threats, such digital strategies may invoke resistance and discontent in the socio-sphere.

Fierce protests against the establishment of an Ebola screening facility of the *Médecins Sans Frontières* in Macenta, Guinea, which was besieged by a hostile crowd in 2014, may serve as an example here. On April 5, urban youth attacked the town’s first EVD clinic and threatened the personnel (Fairhead 2015). The protesters claimed EVD did not exist, or was spread by foreigners. According to screening experts, active resistance to public health measures contributed to the speed and persistence of the epidemic in the region. Such collisions are reminiscent of Gorky’s play discussed above. Protasov was convinced that his science would benefit humankind, but instead of tending his “herd”, he became absorbed in his research, insensitive to what was happening in other strata. In the current era, the focus of attention has shifted from the bio-chemical to the bio-informational level, but the Macenta events reveal similar discrepancies between “non-simultaneous” local and global understandings of health and disease, indicating that, to effectively address emerging viral threats, besides technoscientific rationality, the socio-cultural ambiance should be addressed as well. Whereas viral experts aim to develop effective responses to what they perceive as global risks, local responses rather perceive international organisations such as *Médecins Sans Frontières* as disruptive intruders.

Super-structural (“cultural”) responses reflect discontent in digitalisation as a form of resistance. As Sigmund Freud (1920/1940) already argued, the primary purpose of our sense organs (with their miniature apertures) is to provide protection against overstimulation (*Reizschutz*). This tendency of living organisms to insulate themselves from the outside world already applies to micro-organisms, coaxed inside their cell membranes and evolving anti-viral defence-systems such as CRISPR-cas9. But protection against information overload is also important for our psychic system. It is a life task as important as sensitivity and receptivity (Freud 1920/1940, p. 27). Our sense organs function like small antennae, allowing us to assess minute samples of exteriority. Their primary objective is to safeguard psychic integrity from being overwhelmed by intrusive and disturbing information. From a dialectical perspective, whereas natural sense organs represent the first moment (a situation of information saturation), technoscientific data entail an unsettling intensification of information exposure, resulting in estrangement and discontent. Super-structural instruments for data governance and data ethics (Floridi and Taddeo 2016) are therefore required to regain manageability at a higher level of complexity (negation of the negation).

Rather than discarding Macenta protests as irrational (from the perspective of enlightened technoscience), we should acknowledge the moment of validity at work in instances of suspicion. In the case of the Corona-crises, a similar recoil can be discerned. In 2017, U.S. scientists were invited to visit the Virology Institute of Wuhan: the first Chinese laboratory formally cleared to work with dangerous viruses at biosafety-level 4, the highest level of containment (Cyranoski 2017). While scientists and NGOs outside China voiced worries about escaping pathogens, lab director George Gao argued that, as the world may soon be facing new emerging SARS-like viruses, “we need more contribution from China” (Cyranoski 2017, p. 400). Although experts consider the possibility that COVID-19 originated in Wuhan’s virology lab “improbable”, additional data may still “swing the balance of evidence” (Andersen et al. 2020, p. 452). From a dialectical perspective, the moment of validity in such disputes resides in the fact that viral research, as a global enterprise, after appropriating terabytes of data from bio-citizens using NGS technologies, subsequently confronts these citizens with research outcomes which are beyond their control. NGS technologies supposedly produce digital “commons”: open sources of information beneficial for global humanity. Yet, we also witness appropriation, commodification and privatisation of data on a global scale, giving rise to a digital version of what Marx (1867/1979) referred to as the primary accumulation of capital (Fuchs and Mosco 2015b). Technoscience evolved into a global enterprise where bio-data represent significant value and where data-streams flow towards big data companies, resulting in what Marx and Engels thematised as alienation, for while bio-data become appropriated by these big commercial players, bio-citizens have no say in the means of production (i.e. NGS technologies) and become estranged from the digital commodities (screening programs, therapeutics, theranostics, etc.) they helped to produce, as the dependent consumers of commodities to whose production they actually contributed (Fisher 2015; Nygren and Gidlund 2015).

This also helps to explain disagreements concerning the *Nagoya Protocol* on fair and equitable use of genetic resources, notably the question whether it applies to the sharing of viral pathogens.^8^ This protocol—a supplementary agreement to the *Convention on Biological Diversity* (CBD)—aims to promote fair and equitable sharing of benefits derived from the use of genetic resources. In December 2006, Indonesia challenged the fairness of global preparedness by refusing to share samples of avian influenza A (H5N1). Concerned that viral data became an exploitable resource (comparable to the silks and spices of early modern colonialism), so that wealthier countries would gain access to vaccines while creating cost barriers for others, Indonesia invoked sovereign ownership of viral samples procured in its territory (Gostin et al. 2014). For the *Nagoya Protocol*, pathogen sharing (required to monitor the evolution and spread of viruses) poses a challenge. Global networks such as the WHO *Global* *Influenza Surveillance and Response System* (GISRS) produce and circulate thousands of viral samples yearly. This global network of laboratories aims to foster preparedness by studying and sharing viral samples including H5N1. Dialectically speaking, such networks represent a negation of the negation (as an advanced technoscientific response to disruptive threats), but also a tendency of surveillance capitalism towards exclusively concentrating the global means of knowledge production. The Nagoya Protocol acknowledges ownership claims of Nation States over genetic resources, including viral data. Implementation of the protocol may slow, limit or complicate the sharing of viral pathogens, for instance by requiring case-by-case *Prior Informed Consent* (PIC) agreements. Whether viral pathogens should be included in the Protocol is a matter of dispute because the legal status of viral pathogens is undetermined. The Nagoya protocol considers viruses as genetic resources under the scope of the United Nations’ *Convention on Biological Diversity* (CBD) over which nation states can claim ownership rights (Rourke 2018). The question is, however, whether the biological nature of viruses as global entities concurs with the logic of the Nagoya Protocol. The issue reveals non-simultaneity (contradiction) between the global, viroscientific logic of sample sharing versus governance conceptions based on sovereignty of nation-states. Viruses are global entities, with a fluid identity and capacity to evolve quite rapidly, so that the question who may claim ownership of viral strains remains a difficult issue. These “super-structural” collisions over legal interpretations symptomatically reflect the socio-economic tensions involved. As information becomes subject to commodification, a limited number of global players may claim ownership over massive amounts of data produced by millions of individuals around the globe, who subsequently will have to buy the products of digital commodification on the global market. This explains the clash of language games of sovereign nation states versus the language game of global networks (Lyotard 1983). Both languages exist simultaneously, but represent different modes of production, circulation and commodification. While from a nation state perspective the sharing of pathogen information may be regarded as “bio-piracy” (Cressey 2017), from a global perspective the insistence on specific agreements is a contra-productive mechanism of defence (Gostin et al. 2014). Thus, potentially dangerous viruses reveal the simultaneity of contradictory governance regimes.

The term “preparedness” (Lakoff 2007, 2008, 2017) conveys a specific “logic”, a specific mode of “tending” the global human herd, a specific experience of space and time (Caduff 2014). The present is framed as an episode of suspension against the backdrop of a looming global cataclysm. Preparedness decreases the temporal gap between viral evolution and technoscientific response (Thomas 2014). To keep up with viral mutations, near simultaneity between threats and interventions must be maintained, turning preparedness into a permanent stance (Caduff 2015b). Looming on the horizon is not just the next pandemic, moreover, but also the next audit, so that researchers and policy makers tend to err on the side of precaution (Caduff 2014). The strive for preparedness includes efforts to technologically reproduce and study lethal viruses in vitro (e.g. Wuhan’s biosafety-level 4 institute). Researchers who analyse and publish data concerning potentially lethal viruses, transmit and circulate sensitive information, so that they themselves become the targets of surveillance and biosecurity regulations, notably because of the risk of “dual use” of viral knowledge for nefarious purposes (Swazo 2013). H5N1 became a telling example of how the source of threat may shift from viral pathogens themselves to the *production, transmission and circulation* of viroscientific information. In response to a paper published by viroscientist Ron Fouchier et al. (2013), the Dutch government declared that they should have applied for an export permit before submission, in the light of EU directives on dual use goods. The authors themselves argue that researchers have a public health responsibility to conduct H5N1 virus transmission studies to ascertain pandemic preparedness by understanding the evolution and adaptive repertoires of influenza viruses. Thus, against the backdrop of global interconnectedness, the spread of viral *information* becomes the issue of concern. The paper finally appeared in a special *Science* issue devoted to H5N1.^9^

The H5N1 case also became a source of inspiration for best-selling novelist Dan Brown, whose novel *Inferno* (about a bio-molecular genius who becomes a bioterrorist) contains the following remark: “Just recently, two very respected virologists—Fouchier and Kawaoka—had created a highly pathogenic mutant H5N1 virus. Despite the researchers’ purely academic intent, their new creation possessed certain capabilities that had alarmed biosecurity specialists and had created a firestorm of controversy online” (Brown 2013, p. 452; Zwart 2014). As a result, both researchers became the target of *ad personam* email-messages, which raises issues concerning the ethics of novel writing (whether it is admissible for literary authors active on global podiums to target really existing persons while addressing sensitive issues in viral research). Ultimately, the question is whether the circulation of viral data can be responsibly contained. A lethal viral sample, kept in a laboratory fridge, becomes a biomolecular “omen”, announcing the advent of the next viral threat (Caduff 2014).^10^

In terms of non-simultaneity: while virologic technologies (as global means of knowledge production) evolve at a rapid pace, national oversight infrastructures have difficulties keeping up. Whereas traditional research ethics focusses on the responsibilities of individual researchers (micro-ethics), the challenges involved in global technoscience emerge on institutional or even global policy levels (macro-ethics), focussing on collective strategies for the responsible management of sensitive information (Zwart 2008). Although Fouchier et al. (2013) acknowledge the responsibility of researchers to contribute to global preparedness, this only applies to researchers who work in elite institutional environments where this type of research can be conducted in a safe and responsible manner. Scientists should only conduct research concerning viral threats, they argue, if appropriate facilities, oversight and approvals are in place. In other words, the globalisation of viroscience as a research practice imposes significant ethical, legal and biosecurity requirements, both concerning the base (production and transmission of data) as well as concerning the normative (legal and bioethical) superstructure, to prevent non-simultaneity between virologic technoscience and biosecurity governance. But this may either result in deepening the digital divide (by exclusive elite network of research) or in suspicion against newcomers (as in the case of the Wuhan lab).

## Conclusion

Besides technoscientific prowess, global resilience vis-à-vis disruptive viral threats requires that the super-structural, socio-cultural dimension is duly addressed. A bioethical assessment of normative challenges involved in contemporary viroscience should acknowledge the interpenetration of cultural, technological, economical and demographical transitions. Building on a dialectical materialist (Marxist) perspective, this paper posits emerging viral diseases in a global force field marked by non-simultaneous practices on global, national and regional levels. Rather than seeing bioethics as a neutral vocabulary, moral valuations are entangled with technoscientific and socio-economic structures, while moral convictions reflect modes of knowledge production and circulation. We have seen how hygienic measures, symbolised by disposable items such as plastic gloves, mouth masks and condoms, may challenge cultural or experiential meanings attached to physical proximity in practices such as human-animal interaction and ritual burials. Besides being experienced as offensive (suggesting uncleanliness of the individuals involved), such disposable items symbolise a mode of production, a socio-economic system: the era of globalisation. Collisions on the socio-cultural level are entangled with disruptive transitions on the global socio-economic level. As Marx and Engels (1845/1969) argued, normative ideas (consciousness) are shaped by the practices in which humans are actually involved. Norms and values connected with global, evidence-based practices may collide with norms and values embedded in local practices.

While implementing or imposing evidence-based health policy measures, resistance is often framed as irrational or misguided, but this may actually reflect the logic of a particular techno-economic system striving for dominance, while what is presented as common interest may actually serve the interests of particular networks of actors. Rather than seeing viroscientists and public health experts exclusively as producers of validated knowledge, while framing dissenting views and experiences as non-simultaneous, there are knowledge deficits and ideological biases on the part of technoscientific and global policy experts as well, especially concerning the socio-economic backdrop and socio-cultural implications of the health policies they support or promote. The questionability of regulatory requirements resides in the disruptiveness of the socio-economic conditions they aim to contain. Discontent in evidence-based policy measures may reflect the growing distance between consumption and production of food (“estrangement”) for instance. Non-simultaneity is not necessarily a one-directional process, moreover, as small-scale regional food production, reduction of global mobility and mitigation of the urban–rural divide may actually become part of a more sustainable future. To effectively address the bioethical challenges of emerging viral diseases, we must acknowledge the extent to which normative challenges are connected with processes of production and circulation of food and other bio-items. Working through the normative challenges entailed in concrete viroscientific collisions contributes to the development of a critical and inclusive global bioethics or macro-ethics.

## Footnotes

1 It has been claimed, but also disputed, that G.W.F. Hegel, the father of modern dialectics, and a consistent source of inspiration for Marx and Engels, fell victim to the cholera epidemic in Berlin in 1831.

2 As an anonymous reviewer phrased it, Marxism had become a dead or at least marginalised academic language until financial and ecological crises paved the way for a revitalization.

3 This discovery occurred 1 year after the publication of Bram Stoker’s novel *Dracula* (1897/1993), depicting the spread of vampirism as a quasi-viral infection which threatens a modern metropole (London) after an isolated niche (Transylvania) had been opened up by globalisation (Zwart 2018a). Stoker’s novel describes a collision between rural Transylvania (under the sway of despotic aristocracy) and modern capitalism: two historical conditions which (due to technological innovations such as trains, steamers and telegraphy) suddenly co-exist simultaneously.

4 Cf. “We can look forward with confidence to a considerable degree of freedom from infectious diseases at a time not too far in the future” (Cockburn 1963, p. 1058).

5 *The Siberian Times*, October 1 2016.

6 In his rereading of Marx, Louis Althusser (1962) already addressed this in terms of “unevenness” and “overdetermination”.

7 Norwegian playwright Henrik Wergeland (1835) wrote a play entitled *Indian cholera* in which an English factory governor forces an Indian Raja to reveal the location of a treasure (Schiedermair 2012). Upon opening a shaft where the treasure is allegedly hidden, the governor unleashes cholera in the shape of a demon, affecting India and quickly spreading into Europe.

8 WHO Secretariat (2016) “Implementation of the Nagoya Protocol and pathogen sharing: public health implication. http://www.who.int/influenza/pip/2016-review/NagoyaStudyAdvanceCopy_full.pdf.

9 https://www.sciencemag.org/site/special/h5n1/.

10 In his book *The Monster at Our Door: The Global Threat of Avian Flu*, Davis (2005) declared that the “essence of the avian flu threat… is that a mutant influenza of nightmarish virulence… is searching for the new gene or two that will enable it to travel at pandemic velocity” (p. 8).
